# Therapeutic Strategies to Reduce Burn Wound Conversion

**DOI:** 10.3390/medicina58070922

**Published:** 2022-07-11

**Authors:** Alen Palackic, Jayson W. Jay, Robert P. Duggan, Ludwik K. Branski, Steven E. Wolf, Naseem Ansari, Amina El Ayadi

**Affiliations:** 1 Department of Surgery, University of Texas Medical Branch, Galveston, TX 77555, USA; alpalack@utmb.edu (A.P.); jwjay@utmb.edu (J.W.J.); rpduggan@utmb.edu (R.P.D.); lubransk@utmb.edu (L.K.B.); swolf@utmb.edu (S.E.W.); 2 Division of Plastic, Aesthetic and Reconstructive Surgery, Department of Surgery, Medical University of Graz, A-8036 Graz, Austria; 3 Department of Biochemistry and Molecular Biology, University of Texas Medical Branch, Galveston, TX 77555, USA; nansari@utmb.edu

**Keywords:** burns, wounds and injuries, biologics, inflammation

## Abstract

Burn wound conversion refers to the phenomenon whereby superficial burns that appear to retain the ability to spontaneously heal, convert later into deeper wounds in need of excision. While no current treatment can definitively stop burn wound conversion, attempts to slow tissue damage remain unsatisfactory, justifying the need for new therapeutic interventions. To attenuate burn wound conversion, various studies have targeted at least one of the molecular mechanisms underlying burn wound conversion, including ischemia, inflammation, apoptosis, autophagy, generation of reactive oxygen species, hypothermia, and wound rehydration. However, therapeutic strategies that can target various mechanisms involved in burn wound conversion are still lacking. This review highlights the pathophysiology of burn wound conversion and focuses on recent studies that have turned to the novel use of biologics such as mesenchymal stem cells, biomaterials, and immune regulators to mitigate wound conversion. Future research should investigate mechanistic pathways, side effects, safety, and efficacy of these different treatments before translation into clinical studies.

## 1. Introduction

Since 2009, over 220,000 patients have been admitted for acute burns in the United States, with deaths eclipsing 6700 [1]. Worldwide, an estimated 180,000 patients die from burns each year, predominantly in comparatively resource-poor nations [2]. Acute burns can be induced in response to thermal (liquid, solid, or gaseous agents), electrical, chemical, or radiation exposure. Acute care of burn patients is costly, and survivors may suffer long-term disabilities secondary to chronic wounds, contractures, or amputations. Still, the initial burn management, in the form of aggressive fluid resuscitation and early excision and grafting, is not only lifesaving but can ameliorate some of the long-term outcomes in burn survivors. Dampening the initial hypermetabolic status is known to minimize the amount of nonviable tissue and improve outcomes, including reduced healing time and scarring [3,4].

**Figure 1:**
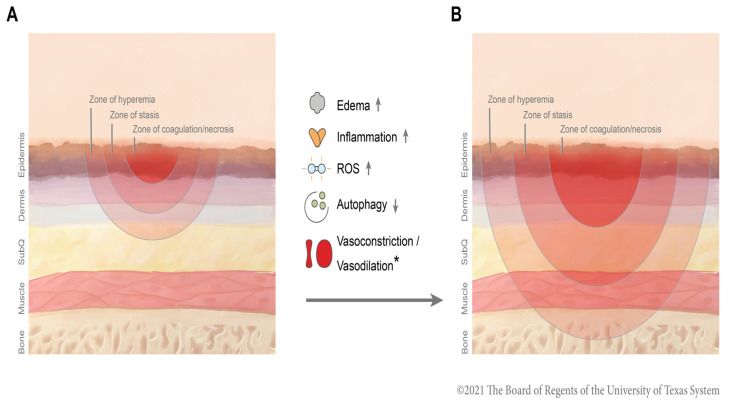
**Mechanisms of burn wound conversion:** Various mechanisms are involved in burn wound conversion from a second partial-thickness (**A**) to a full-thickness burn (**B**), including increased ROS generation, proinflammatory cytokines generation, increased edema, and reduced autophagy. * Due to contradictory data in the body of the literature, evidence for both increased and decreased wound perfusion exists, as discussed in this review.

Excision and grafting are the foundations of burn care where nonviable deep partial-thickness and full-thickness burn wounds are promptly debrided and subsequently covered. More superficial wounds are typically dressed and left to heal spontaneously, and reepithelization occurs from appendages retained within the viable dermis. Burn wound conversion refers to the phenomenon whereby partial-thickness burns that appear to retain the ability to spontaneously heal may convert into deep partial-thickness or full-thickness wounds in need of excision. Jackson was the first to describe this process when classifying burn injury zones that present different degrees of tissue damage. These zones of damage usually fluctuate between a zone of full-thickness burn (zone of coagulation) and a surrounding area with partial-thickness and deep-partial thickness burn (called the zone of ischemia or stasis) (Figure 1). The zone of stasis is the area that purportedly hangs in the balance that may be salvaged, minimizing the amount of tissue in need of excision, and improving overall wound healing outcomes [5,6].

Previous studies have addressed the process of burn wound conversion and treatment strategies to prevent the zone of stasis from becoming necrotic [7,8,9,10,11]. While no current treatment can definitively stop burn wound conversion, attempts to slow tissue damage remain unsatisfactory, justifying the need for new therapeutic interventions. Studies show that targeting at least one of the molecular mechanisms underlying burn wound conversion, including ischemia [12], inflammation [11,13,14], apoptosis [12,15,16,17], generation of reactive oxygen species [10,16], hypothermia [9], wound rehydration [14], and more recently autophagy [8,15,18], have the potential to reduce wound conversion. This suggests the need for pleiotropic agents that can target various mechanisms involved in burn wound conversion at the same time.

We searched the PubMed and Google Scholar databases for primary research articles published between January 2012 and March 2022 with combinations of keywords including “burn wound conversion,” “pathophysiology,” “wound healing,” “therapy,” “regulation,” and “biologics” in the title, keywords, or abstract sections to identify clinical or basic science investigations. We have considered both clinical and animal studies. However, there remains a dearth of evidence for human trials with interventions targeting burn wound conversion specifically; therefore, most studies presented herein utilized basic science animal models to test potential novel treatments or identify previously unknown mechanisms in burn wound conversion. In limited cases, we have included seminal research articles (prior to 2012) for historical perspective and relevance in burn wound conversion research. Overall, we aimed to give an overview of the pathophysiological pathways and new therapeutic approaches involved in burn wound conversion throughout the last decade, which may allow physicians to maximize the area of tissue viability and improve long-term outcomes in burn survivors.

We have chosen to review the current literature focusing on those burns that require acute professional medical care (i.e., burns requiring hospital admission). However, it is noteworthy to briefly mention pre-hospital care and first aid for burns in the context of wound conversion. As Goodwin eloquently demonstrates, currently there remains a lack of consensus among government agencies, international burn societies, hospital burn units, and even United Nations health councils on the 2021 published recommendations for emergency first aid care and treatment of burn wounds [19,20]. Many expert bodies recommend the use of cooling deep partial-thickness wounds; however, there remains tremendous heterogeneity in the cooling method suggested. Furthermore, a growing majority of authoritative councils recommend withholding cooling altogether for burn first aid. Additionally, there are discrepancies in the use of emergency hydrogel application, which may be further exacerbated by conflicts of interest with commercial entities that have contracts to supply first aid material to emergency services [19,20]. With such variation in burn emergency first aid coupled with a dearth of primary literature and correlational studies on the effects of burn first aid on wound conversion, a major research focus in this area is warranted.

## 2. Inflammation and Burn Wound Conversion

The negative effects of prolonged inflammation on burn wound conversion are well established, and a variety of factors are involved in the protracted postburn inflammatory response. The inflammatory response is the first critical step in healing a burn wound. Inflammation is necessary, and perturbations, including a muted response or over-response, during this critical phase may be responsible for the process of wound conversion; thus, therapies targeting the inflammatory phase of wound healing have been of great interest to burn researchers. Cellular components include cytokine production, ROS generation (discussed below), and delayed apoptosis or senescence of inflammatory cells [3,4,21]. A persistent presence of inflammation markers such as C-reactive protein and complement factors was reported in burn wounds [22]. In a mouse model of comb burn, neutrophil sequestration and activation induce microvascular damage, which leads to secondary tissue damage and necrosis [23].

The standard of care in burns involves the mechanical wound debridement of eschar and slough followed by the application of biological or synthetic dressings [4]. However, novel treatment approaches are now targeting detrimental inflammatory mediators and signaling pathways. Among those, TNF-α is one of the inflammatory mediators that is significantly elevated in burn wounds and can be targeted using TNF- α inhibitors. It is produced early after burn, in both the coagulation zone and the zone of stasis [23]. In a rat comb burn model, our group showed that shortly after burn injury, both the coagulation zone and interspaces show increased inflammatory cell infiltration around blood vessels and interstitial spaces in hypodermal and sub-muscular areas [24]. This effect was attenuated with early treatment using a metal chelator in combination with methylsulfonylmethane [24,25]. Another rat model showed promising results when conjugating TNF- α antibodies with hyaluronic acid (HA) in a topical application [26]. When conjugated to HA, TNF- α antibodies prevent necrosis and decrease inflammatory markers more efficiently than TNF-α alone or anti-TNF-α mixed, but not conjugated, to hyaluronic acid [26].

In another study, Sun and colleagues showed that HA-conjugated with anti-IL-6 and TNF-α antibodies not only decreased inflammation but significantly reduced burn wound progression when applied to a rat partial-thickness burn model [27]. Other studies targeting TNF-α have investigated Cerium nitrate as a potential therapeutic approach. In a preclinical rat model, Cerium nitrate was shown to attenuate burn wound conversion by reducing TNF-α levels [28] and leukocyte transmigration [29]. These findings suggest that reducing TNF-α may be a good strategy to attenuate burn wound conversion [28,29]. However, to the best of our knowledge, there have yet to be any studies confirming the clinical effectiveness of TNF-α inhibition in reducing burn wound conversion.

While Interleukin-6 (IL-6) is a proinflammatory cytokine that plays an important role in the hypermetabolic response to a burn, studies using antibodies to target IL-6 showed no significant reduction in burn wound conversion [4]. Research continues to target the protracted postburn inflammatory phase, and a recent study used an oil-in-water nanoemulsion formulation (NB-201) containing benzalkonium chloride to reduce burn wound conversion in a porcine burn model [30]. This study indicated that the production of inflammatory mediators and sequestration of neutrophils was inhibited by NB-201. They also showed that NB-201 prevention of wound conversion from a partial- to a full-thickness burn was associated with a concurrent decrease in dermal inflammation [30]. The nucleotide-binding oligomerization domain-like receptor family, pyrin domain containing 3 (NLRP3) inflammasome also plays an important role in the inflammatory response after a burn. In a preclinical rat model, Xiao et al. demonstrated that burn-induced inflammasome activation and inflammatory response in wounds may be associated with burn wound progression. In their study, treatment with 3,4-methylenedioxy-β-nitrostyrene inhibited NLRP3 inflammasome activation, ameliorated burn wound progression, and promoted wound healing [31].

Local administration of corticosteroids to prevent vertical burn progression has been recently investigated. Despite its anti-inflammatory components, topical application of steroid cream could not prevent vertical burn wound conversion in a deep-partial thickness burn animal model. A systemic non-pharmacologic approach is the utilization of hypothermia. Interestingly, various studies have shown that systemic hypothermia downregulated inflammatory mediators and decreased vertical burn progression [9]. Additional data show that localized cooling mitigated cellular damage in the wound bed compared to ambient temperatures [32]. However, contradictory studies show that hypothermia can also upregulate inflammatory mediators such as TNF-alpha, which may delay wound healing and contribute to conversion [33]. There is even further conflicting evidence suggesting that a locally warm environment may prove beneficial in the healing of burn wounds. Early studies by Tobalem et al. (2013) showed that burn wound conversion in rats can be halted with the use of warm water (37 °C) directly on the wound bed when compared to cold water (17 °C) [34]. The investigators concluded that the warmer healing environment contributed to greater microvascular perfusion and thus, greater tissue survival [34]. Further complicating the issue, some commonly adopted international guidelines recommend using cold water as first aid in burn care—arguing that cooling the partial thickness burn wound reduces cell death and decreases wound size [35]. However, the role of inflammation was not addressed in these burn wound management studies conceding contradictory evidence on the best treatment strategies concerning warming or cooling the wound. This all suggests that targeted treatment approaches are preferable compared to the utilization of systemic non-pharmacologic means. An illustration of the role of inflammation in clinical trials will confirm the efficacy of these treatments.

## 3. Ischemia

Impaired perfusion of the burn wound often occurs secondarily due to hypovolemic shock. This leads to cell and tissue death and, consequently, to wound conversion [36]. Ischemia is known as one of the major contributors to burn depth progression in the zone of stasis. The etiology includes thrombosis, vasoconstriction, and edema [4,36]. From a pathophysiological standpoint, severe burn causes vasoconstriction and fluid loss due to increased vascular permeability. This leads to impaired tissue perfusion and consequently may result in tissue necrosis and a deeper wound. Conversely, hypervolemia is also a known driver of wound conversion. A systemic approach to counter impaired perfusion hypovolemia is fluid resuscitation. It is a standard-of-care milestone in acute burn care and plays a major role in the management of burned patients and is essential to maintaining tissue perfusion [37]. Several studies have shown that inadequate fluid resuscitation exacerbates the tendency toward burn wound deepening [38]. In a rat model of fluid resuscitation, Kim et al. showed that conversion of partial- to full-thickness burns is hastened when fluid resuscitation fails to provide adequate flux, or perfusion, to burn wounds [39]. Similarly, in a review of pediatric burn resuscitation, Carvajal argued that restoring and maintaining perfusion pressures maximally oxygenates injured and non-injured tissues [40] and, as such, promotes spontaneous healing while minimizing wound conversion and bacterial colonization. One of the local causes of burn wound conversion is desiccation; it compromises the perfusion of burn wounds via shifts in intravascular and interstitial fluid volumes, leading to increased tissue necrosis and greater eschar formation. The decrease of intracellular fluid volumes alters burn wound hemostasis in a manner that ultimately reduces intravascular volume. Furthermore, it inhibits oxidative metabolism of partially burned tissue [41]

In recent years, the implementation of systemic erythropoietin (EPO) in burns and wound progression has been widely studied. EPO is an antiapoptotic, anti-inflammatory, and immunomodulatory cytokine [42] with properties that increase vasodilation and induce angiogenesis. Studies have shown that administration of 500 units EPO/kg once a day for 5 days, starting 45 min after the burn, decreases burn depth and, when combined with cold water, increases nitric oxide synthase expression and decreases inflammation [7,42,43]. Furthermore, animal studies explored time and dose dependence in the administration of EPO. They showed that administration of low-dose EPO, as introduced above, prevents secondary burn wound progression. Immediate utilization of EPO within 45 min, but not later than 6h postburn, has beneficial effects on burn wound progression [7,44]. Günter and colleagues investigated EPO in a randomized clinical trial and showed lower morbidity in severely burned patients receiving EPO, suggesting pro-regenerative effects of EPO in burned patients [45].

Early burn wounds are significantly impacted by cellular injury, which probably results from the loss of cell membrane integrity, the increase of inflammatory mediators, and peroxidation insults. Therefore, some investigators have examined the effects of Poloxamer 188 (P188) on burn wound conversion [46]. P188 is a unique copolymer that prevents endothelial cell injury and reduces leukocyte adhesion. The effect of P188 on reducing the conversion of deep-partial thickness burn wounds was studied in the early stage after burn in a rat model. Topical administration of P-188 (200mg/kg every 24 h) significantly reduced secondary burn wound conversion. In this study, improved tissue survival correlated with increased Na-KATPase decreased malondialdehyde/oxidative damage and reduced inflammation in the zone of stasis [46]. Other agents have been studied to target endothelin, a vasoconstrictor peptide that regulates systemic blood flow and is elevated in severe burns, causing ischemia and subsequent tissue necrosis [47]. TAK-044, a nonselective endothelin A, and endothelin B receptor antagonist were explored in a rat burn model in 1997 [48], and one administration of TAK-044 (1 mg/kg) significantly reduced the area of necrosis [48]. Even with these promising results, the administration of TAK-044 for the treatment of secondary burn wound conversion has not been studied since. Nicorandil, an adenosine triphosphate-sensitive K + channel opener, is another agent that has been explored in burn wound conversion. In a preclinical rat comb burn model, Nicorandil increases tissue survival in the zone of stasis by attenuating ischemia-reperfusion injury. [49] Heparin, which is known as an anticoagulant agent, also possesses anti-inflammatory and anti-angiogenic properties, as well as a capacity for promoting wound healing. In a review of the literature, Saliba et al. reported that heparin relieved pain, prevented clotting and inflammation, restored blood flow, and promoted wound healing in burn patients. [50] All of these studies highlight the ongoing need for further research that targets postburn ischemia to prevent detrimental wound conversion.

## 4. T Cell and Macrophages in the Burn Wound

T cells are derived from fetal thymic precursors and are classified into various subtypes that play an important role in the innate and adaptative immune response. Among T cells, the gamma delta (γδ) T cells are a minor subpopulation of resident lymphocytes restricted to the skin [51,52]. These cells assume a dendritic morphology in healthy skin and constitutively produce low levels of cytokines that contribute to epidermal homeostasis [52]. γδ T cells express the canonical T cell receptor (TCR) that is only expressed in resident T cells [52]. When skin is wounded, an unknown antigen is expressed on damaged keratinocytes. Neighboring γδ T cells then contribute to wound healing by local production of epithelial growth factors and inflammatory cytokines [52]. Wound healing is impaired in the absence of γδ T cells [52,53]. A preclinical study showed that burned mice lacking γδ T cells have a notable defect in the early inflammatory phase of wound healing and reduced infiltration of inflammatory cells into the burn wound [54]; thus, the wounds could not benefit from epithelial growth factors, which prevented healing and promoted an environment for conversion. This adds more knowledge to the concept of regulated inflammation. Previous studies by Toliver-Kinsky et al. have shown that mice deficient in the T-cell receptor are resistant to early inflammation following cecal ligation and puncture [55]. Using the burn wound infection mouse model, the same investigators showed that dendritic-cell-deficient mice displayed delayed wound healing, suppressed early cell proliferation, reduced formation of granulation tissue, and decreased angiogenesis [42], which may contribute to wound progression and conversion. Infiltrating T cells following a severe burn are also the main producers of proinflammatory cytokines such as TNF-α and IL-6, which are known drivers of wound burn conversion through necrotic expansion [56].

With this knowledge, other investigations have attempted to minimize these proinflammatory cytokines in the burn wound microenvironment to mitigate wound progression. In a recent series of investigations, we have shown that metal chelation, topically applied to porcine and rodent full-thickness burn wounds, significantly reduces IL-6 and TNF-α expression during the initial phase of wound healing and minimizes wound conversion [25,57,58]. These promising results show that temporal targeting of proinflammatory T cell cytokines may be an important strategy for limiting burn wound conversion.

Macrophages have emerged as key players in the progression of wound healing and thus, may play a critical role in burn wound conversion [59,60]. They are classified as proinflammatory “classically” activated M1 macrophages and the “alternatively” activated M2 macrophages. Macrophages can polarize in these two phenotypes upon activation by various stressors [59,61]. The M1 phenotype is associated with inflammation and recruitment to the wound area. The M2 phenotype is associated with proliferation and tissue repair [62]. Furthermore, non-functional or dysfunctional M2 macrophages can accumulate and aggregate in chronic non-healing wounds [60], pressing the importance of synchronized and timely M1 to M2 polarization for optimal wound healing. Macrophage polarization towards an M2 phenotype was shown to be mediated by the AMPK/mTOR/NLRP3 31 and the AMPK-NF-κB pathways [61]. Activation of mTOR, in this case, could be important for the induction of autophagy to eliminate dead, aggregated, dysfunctional proteins and organelles to promote cell survival, provide a cellular mechanism to maintain homeostasis, and provide a potential mechanism to halt a converting wound (see Section 5—Autophagy). While these studies address the role of TCR activation in the early inflammatory response to a burn, macrophage regulation of T-cell activation and its role in wound conversion is poorly understood.

## 5. Autophagy

Autophagy is a form of cell death that is a tightly regulated and conserved biogenic process used by tissues during critical times of energy depletion. Using autophagy, cells can respond to severe injuries such as burns through lysosomal proteolysis by recycling defective or misfolded proteins and depleted organelles; this process can rapidly replenish amino acids to maintain tissue homeostasis. Although autophagy and apoptosis are conceptually considered to be independent physiological processes with opposite results, the two mechanisms are coupled in many ways. Autophagy and apoptosis have overlapping signaling pathways that contain major elements such as p53 and BCL-2 family proteins and some essential pro-autophagic proteins initially cleaved and activated by apoptotic caspases [63,64,65].

While the role of apoptosis in wound conversion is established [66], the role of autophagy in this process has recently started to gain some attention. Autophagy was shown to limit and, in some cases, halt burn wound conversion. Early studies showed apoptotic-associated proteins played a significant role in post-burn autophagic processes as well [17]. In their murine brass rod burn model, investigators showed for the first time that beclin-1 and LC3 triggered autophagy of dermal fibroblasts in a deep partial-thickness burn instead of directly activating apoptosis. Results indicated that a more enhanced autophagic profile could slow wound progression and protect against conversion to a deeper wound [67]. Indeed, a preclinical model from Xiao and colleagues showed that intervention with Rapamycin, a known autophagy activator, after a severe burn reduced proinflammatory cytokines and significantly enhanced wound bed autophagy. The Rapamycin-induced autophagic response blunted conversion to a deeper wound compared to standard of care controls [18].

More recent studies elucidate autophagic signaling pathways and confirm previous investigations. In a comb scald model, Guo et al. (2020) showed that post-burn tissue hypoxia significantly increased HIF-1α expression as expected [68]. Typically, increases in HIF-1α result in severe oxidative cellular damage. Their results showed that increased HIF-α subsequently activated BNIP3 and PARKIN, a regulator of the outer mitochondrial membrane and a ubiquitin ligase, respectively, resulting in rapid mitochondrial degradation or mitophagy. Although this would be considered detrimental if left unchecked, here, the mitophagy was protective and inhibited further wound progression in the murine model [68]. These data suggest that post-burn temporal targeting of HIF-1α may be beneficial to restoring wound bed homeostasis.

Using the mice burn model, Han and colleagues (2020) demonstrated that a major histone deacetylase SIRT1 is significantly upregulated after a severe burn [69]. The postburn SIRT1 increase was a critical activator of wound macrophage autophagy and significantly decreased the expression of proinflammatory cytokines IL-1, -6, -10, and -18, which ultimately attenuated the post-burn inflammatory phase and halted wound conversion. To confirm these findings, subsequent SIRT1 inhibition abolished the autophagic response, and the burn wound continued to progress [69]. Future studies will continue to add to our understanding of the role of autophagy in burn wound conversion and should be a major clinical focus as research advances.

## 6. Reactive Oxygen Species

Oxygen supply is critical to normal wound healing. Disruption of oxygen delivery can lead to the formation of reactive oxygen species (ROS), both non-free radicals and free radicals, which are known to interfere with angiogenesis, dermal fibroblast proliferation, and wound re-epithelialization [70]. After a severe burn, oxygen-free radicals’ superoxide (O2•-) and hydroxide (OH•-) are generated as a direct result of thermal injury, tissue necrosis, mitochondrial dysfunction, and phospholipid metabolism. Post-burn activation of NADPH oxidase (NOX), xanthine oxidase (XO), and superoxide dismutase (SOD) can generate the non-free radical hydrogen peroxide (H2O2) species as well. ROS has long been regarded to be a primary mediator in post-burn wound healing, and balanced ROS generation has several beneficial effects [71,72]. Studies have demonstrated that ROS acts as a chemotactic stimulus for wound neutrophils [73] which provides crucial immunological surveillance, can have direct bactericidal effects [74], and plays a pivotal role in cellular adhesion [75]. Moreover, recent animal studies have also shown ROS functions to initiate keratinocyte proliferation and migration—a critical phase of cutaneous wound healing [76]. Nonetheless, excessive ROS production and dysfunctional ROS elimination or sequestration drive cutaneous oxidative stress and protract optimal wound healing. With this understanding, most of the current research now targets post-burn ROS with antioxidant agents to restrict its most damaging effects and limit burn wound conversion.

Antioxidant agents such as N-acetylcysteine and curcumin have long been used to prevent ROS tissue damage and are now being used frequently with success. The acetylated variant of cysteine, N-acetylcysteine (NAC), is used to produce the endogenous and ubiquitous antioxidant enzyme glutathione and has been used with some success in the clinic to treat chronic inflammatory diseases such as COPD and pulmonary fibrosis [77]. NAC was also explored in post-burn therapeutics. In a full-thickness mouse burn model, Landriscina and colleagues demonstrated that NAC attenuated wound expansion and accelerated wound healing by advancing the proliferative phase of wound healing and increased collagen deposition [78]. In another murine model, Saputro et al. showed that NAC administration immediately after injury reduced the necrotic zone and limited tissue damage compared to controls by attenuating the overall ROS burden in the wound [79]. The beneficial effects of NAC on wound healing and reduction of ROS generation in the zone of stasis were corroborated in various studies [80,81,82]. Other treatments such as curcumin have also shown clinical benefits in improving post-burn wounds as well. Produced from the roots of the *Curcuma longa* plant, curcumin has been used in world cuisine as a traditional spice in many curries. Early research showed that curcumin has tremendous antioxidant properties [83,84,85], and it is currently gaining traction as a post-burn therapeutic as well. Several lines of research have demonstrated curcumin’s effectiveness. Seyhan (2019) used emulsified curcumin oil on rats with partial-thickness burns and showed improved wound angiogenesis and decreased fibrosis compared to control treatments [86]. Using a partial-thickness burn mouse model, Sajjad and colleagues showed significant improvement in various clinical parameters, such as vascularization, wound granulation, and re-epithelialization, attributing their findings to curcumin’s antioxidant properties [87]. Other studies showed that curcumin-treated adipose-derived stem cells increased keratinocyte proliferation and migration when used to treat cutaneous wounds in rats [88]. Curcuma also promotes faster healing and reduced wound conversion in murine burn models [89].

More recently, other compounds have been proposed to reduce ROS generation and limit burn wound conversion. Using the brass comb model, our group has shown that metal chelation attenuates oxidative stress, inflammation, and vertical burn wound progression in the Yorkshire pig [25] and the rodent model [24,58]. Zhang and colleagues recently developed a hydrogel with embedded iron-coordinated superoxide dismutase and Vitamin E, which they applied directly to the deep-partial thickness burns of Balb/c mice. Functional, biochemical, and histological analyses showed that the antioxidant hydrogel significantly decreased wound ROS, reduced wound progression, and promoted faster wound repair [90]. In a similar study, Yang also demonstrated that a carboxymethyl chitosan hydrogel successfully and almost completely reduced ROS generated by partial-thickness wounds in rats and significantly improved in vivo wound repair parameters [91]. A recent study on rats has also shown that intradermal injection of the redox dye, methylene blue, can reduce necrosis progression in ischemic perilesional areas suggesting an alternative treatment to reduce burn conversion [92]. These data indicate the therapeutic potential of antioxidants to improve healing outcomes and reduce burn wound conversion.

## 7. Other Treatment Approaches

Hyperbaric oxygen therapy is widely used in the management of chronic wounds [93,94] and was implemented in the treatment of wound healing after burn as well as secondary burn wound progression. Hyperbaric oxygen therapy demonstrated anti-edematous, anti-inflammatory, and anti-hypoxic effects. Hyperbaric oxygen was shown to increase re-epithelization and inflammatory cell migration in a rabbit deep-partial thickness burn model [95]. In Sprague-Dawley rats, hyperbaric oxygen therapy showed beneficial effects at the cellular level and reduced progression of the zone of stasis to necrosis in the first 24hrs after burn [96]. Despite these beneficial effects of hyperbaric oxygen, the special chamber requirement to achieve adequate treatment [96] makes it more feasible for smaller superficial burns rather than severely burned cases. To date, no randomized clinical trials have proved the efficacy of hyperbaric oxygen therapy in preventing burn wound conversion.

The use of a bromelain-based Gel dressing (Debrase) has been studied in the context of enzymatic eschar removal [97]. In a porcine burn model, Singer et al. investigated the effects of Debrase on the zone of stasis by comparing early Debrase application to a control group and a vehicle-only containing gel. In the early phase after the application (4 h), the authors showed that Debrase led to improved dermal collagen structure, decreased microvascular thrombosis, and follicular necrosis compared to the control [98]. Furthermore, they showed that after 48 h, Debrase^®^ reduced the number of unburned interspaces that showed full-thickness burns in comparison to the control group.

Mesenchymal stem cells (MSC) are also used in the treatment of burn wound conversion. While the beneficial effects of MSCs on wound healing are well established [99], some studies showed beneficial effects of MSCs on the survival of the zone of stasis. Singer et al. investigated the systemic administration of MSC in a brass comb model. They showed that IV administration of MSC reduced the area of necrosis by approximately 20% [100]. Using the same model, Oksuz et al. showed that subcutaneous injection of MSCs in the zone of stasis lowered apoptosis counts and increased tissue perfusion and the percentage of vital tissue [8]. In a recent study, Abbas et al. confirmed the previously mentioned findings in a rat brass comb model and explored the mechanisms by which MSCs prevent burn wound conversion. They showed that MSCs reduce neutrophil infiltration, proinflammatory cytokine production, and upregulation of anti-inflammatory cytokines [101]. The same study showed reduced ROS generation, increased angiogenesis, and improved tissue perfusion with MSCs, suggesting the involvement of these mechanisms in MSC-reduced apoptosis in the zone of stasis [101]. When comparing MSCs from various sources, the same group showed that bone marrow, adipose tissue, and dental pulp may serve as universal MSCs donor sources for the reduction of burn wound conversion. Despite these excellent findings, further preclinical studies are required to address questions about the standardization of cultures, dosage, and application methods that will achieve efficacy before translation into clinical studies [102].

Emerging clinical data show alternative approaches for the prevention of burn wound conversion. A retrospective study analyzed hospital records of burn survivors treated with the HMG-CoA reductase inhibitor atorvastatin for any adverse events or life-threatening lab abnormalities during their hospital stay [102]. Atorvastatin was not associated with any adverse effects or lab irregularities, supporting the safety of studying its effects on burn wound conversion in clinical studies [103]. A preclinical study using the Yorkshire pig model showed that atorvastatin treatment enhances graft take, improves wound re-epithelialization, and accelerates neutrophil resolution [104], which may reduce burn wound conversion.

Another therapeutic approach to burn wound conversion is the use of negative pressure wound therapy (NPWT). NPWT has been shown to improve wound perfusion, reduce edema, and accelerate the formation of granulation tissue through various overlapping physical and physiological processes. NPWT has been used for over a decade to treat diabetic ulcers with success and demonstrates a good safety profile, but its use in treating burn wound conversion is less studied. A review of the literature by Dumville et al. in 2014 showed that most clinical studies on NPWT in burns have a high risk of bias, and not enough evidence was available to determine the efficacy of NPWT in the treatment of partial-thickness burn wounds [105]. However, a recent report highlighted two cases of electrical burns that were treated with NPWT in the acute and reconstructive phases; results showed quicker granulation tissue formation and significant improvement in graft take [106], both of which are encouraging indicators for reducing the possibility of conversion from a partial thickness wound to a full thickness wound. Although these results are limited to two patient cases, the report offers promise for post-burn NPWT and has propelled the initiation of larger clinical trials to examine its efficacy [107].

## 8. Conclusions

Regardless of the treatment approach, an intervention for burn wound progression must occur within the first 72 h after injury. Burns can range from superficial to full-thickness burns. In the absence of appropriate treatments, partial-thickness burns can progress and become deep-partial thickness or even full-thickness burns with necrotic tissue. As summarized in Table 1, various mechanisms are involved in burn wound conversion, suggesting the need for multimodal therapeutic approaches. Attenuation of wound conversion will reduce the incidence of wound infection, accelerate wound healing, improve skin function, and prevent scar formation. Our review shows recent advancements in therapies targeting different pathways, such as reducing inflammation and ROS, increasing perfusion, and elucidating the role of autophagy in this process. Advanced studies have turned to biologics, as is the case of mesenchymal stem cells, biomaterials, and immune regulators. Future research should investigate pathways, side effects, and efficacy of different treatments to translate these findings to patients. Furthermore, there is an increased need for different imaging modalities that may better characterize and predict burn wound depth and assist in clinical decision-making.

## Author Contributions

Conceptualization and methodology, A.P., A.E.A.; writing—original draft preparation, A.E.A., A.P., J.W.J., R.P.D.; writing—review and editing, A.E.A., J.W.J., S.E.W., L.K.B., N.A.; supervision, A.E.A.; All authors have read and agreed to the published version of the manuscript.

## Figures and Tables

**Table 1:** Therapeutic approaches targeting various mechanisms of burn wound conversion.

Target	Pathway	Agent	Reference
**Inflammation**	-TNF-α	-Metal chelator in combination with methylsulfonylmethane;	[24,25]
		-Conjugated TNF- α antibodies with hyaluronic acid (HA) in a topical application;	[26]
		-Hyaluronic acid-conjugated anti-IL-6 and TNF-α antibodies conjugated antibodies	[27]
	-TNF-α and leukocytes transmigration	-Cerium Nitrate	[28,29]
	-IL-6	-Antibodies vs. IL-6	[4]
	-Inflammatory mediators and sequestration of neutrophils	-Oil-in-water nano emulsion formulation (NB-201) containing benzalkonium chloride	[30]
	-NLRP3 inflammasome activation	-3,4-methylenedioxy-β-nitrostyrene	[31]
	-Neutrophil infiltration, proinflammatory cytokine production, and upregulation of anti-inflammatory cytokines	-Mesenchymal stem cells	[101]
**Ischemia**	-Impaired tissue perfusion	-Fluid resuscitation	[37,38,39,40]
	-Desiccation	-Allogeneic keratinocytes cultured on acellular xenodermis	[41]
	-Increase in nitric oxide synthase expression with decreased inflammation	-Erythropoietin	[7,42]
	-Cellular Injury	-Poloxamer 188 (P188)	[46]
	-Endothelin A, and endothelin B	-TAK-044	[48]
	-Adenosine triphosphate-sensitive K + channel	-Nicorandil	[49]
**T-cells and macrophages**	-IL-6 and TNF-α expression	-Metal chelation	[24,58]
**Reactive Oxygen Species (ROS)**	-Reduction in ROS	-N-acetylcysteine (NAC);	[78,79,80,81,82]
		-Curcumin;	[87]
		-Curcumin-treated adipose-derived stem cells	[89]
		-Metal chelation	[25]
		-Hydrogel with embedded iron-coordinated superoxide dismutase and Vitamin E;	[90]
		-Carboxymethyl chitosan hydrogel,	[91]
		-Methylene blue intradermal injections	[92]
		-Mesenchymal stem cells	[101]

## Data Availability

Not applicable.
